# The Royal British Nurses' Association and the College of Nursing, Limited

**Published:** 1917-03-31

**Authors:** Herbert J. Paterson

**Affiliations:** Medical Hon. Secretary, R.B.N.A. London, W.


### THE ROYAL BRITISH NURSES' ASSOCIATION AND THE COLLEGE OF NURSING, LIMITED.

"THE SECRET COMPACT" REVEALED.

*To the Editor of* THE HOSPITAL.

SIR,—At the Special General Meeting of the Royal British Nurses' Association held on January 18 last, I referred to correspondence which had passed between the Hon. Arthur Stanley and myself with regard to certain conditions incidental to the proposed amalgamation of the Royal British Nurses' Association and the College of Nursing, Ltd. In certain quarters this correspondence appears to be regarded as a deep-laid scheme for depriving the nursing profession of some cherished birthright, and has been referred to as a "secret pact."

As a matter of fact there has been no secrecy at all. At the Special General Meeting I referred to every point in the correspondence except one, which at the time escaped my memory. There is nothing to conceal. Her Royal Highness the Princess Christian, appreciating the value of Miss Macdonald's work and the feelings entertained towards her by the members of the Association, was pleased to instruct the honorary officers to safeguard Miss Macdonald's position. As this could not be accomplished fully in the By-laws or in the Agreement, I was requested by the Executive Committee to see Mr. Stanley and discuss this and other points about which strong representations had been made to the Executive Committee by many of our members. I embodied in a letter the various conditions discussed, Mr. Stanley concurred with them, and subsequently, in reply to a letter drafted by our solicitor, Mr. Sydney Pitt, the conditions were accepted by the Hon. Arthur Stanley and Sir Cooper Perry. This is the whole story.

While the Executive Committee do not recognise the right of any individual member to demand, as a matter of right, as Miss Hawkins has done, a copy of any correspondence passing between them and any other person or persons, such correspondence being obviously confidential, nevertheless the Executive Committee realise that this matter is one of interest to the members of the Association, and that its publication will show how little justification there is for the insinuations which have been made, and so have authorised me to publish it, a course to which the Hon. Arthur Stanley has given his consent. I may perhaps be pardoned for stating that not a single member of the Association, other than Miss Hawkins, has inquired either verbally or in writing as to this "mysterious pact." No more signal proof could be afforded of the confidence which the members repose in their honorary officers and Executive Committee. I venture to hope, therefore, that you will be able to find space for the correspondence, a copy of which I enclose herewith.—I am, Sir, yours faithfully,

HERBERT J. PATERSON,
Medical Hon. Secretary, R.B.N.A.

London, W., March 23, 1917.

